# Supplementary material for: Zero-shot interpretable phenotyping of postpartum hemorrhage using large language models
Source: NPJ Digit Med. 2023 Nov 30;6:212. doi: 10.1038/s41746-023-00957-x (PMC10689487; doi:10.1038/s41746-023-00957-x)
Supplement: Supplementary file 1 — Supplementary Information [file 41746_2023_957_MOESM1_ESM.pdf]

Supplementary Information for

Zero-shot Interpretable Phenotyping of Postpartum Hemorrhage Using Large Language Models

Concept Type	Concept	Annotation instructions
Medication	Carboprost	carboprost (hemabate) given for inadequate uterine tone, example ’hemabate’
	Methylergonovine	methylergonovine (methergine) given for inadequate uterine tone, example ’methergine’
	Misoprostol as a utero-tonic	misoprostol (cytotec) given for inadequate uterine tone after delivery; do not annotate when given to induce labor (in this case, it is administered before delivery); example ’cytotec’
Procedure	Bakri balloon	Bakri balloon or hemostatic balloon inserted in the uterus to stop bleeding, example ’Bakri balloon’
	Dilation and curettage	procedure performed after delivery to remove retained products of conception; examples: ‘Dilation & curettage’, ‘D&C’, ‘D&E’, ‘Dilation and evacuation’, ‘D and E’.
	Manual extraction of placenta	manual extraction/evacuation/removal of the placenta or retained products of conception
	O’Leary sutures	O’Leary sutures performed on the uterus to stop bleeding, example ’O’Leary’
	Hysterectomy	hysterectomy, or surgical resection of the uterus; examples ‘C-hyst’, ‘Cesarean hysterectomy’, ‘hyst’.
Transfusion	Fresh frozen plasma	transfusion of fresh frozen plasma, examples ’ffp’, ‘FFP’, etc.
	Cryoprecipitate	transfusion of cryoprecipitate, examples ’cryo’, ‘CP’, etc.
	Platelets	transfusion of platelets, examples ’plt’, ‘Pt’, etc.
	Packed red blood cells	transfusion of packed red blood cells, examples ’prbc’, ’rbc’, etc.
Problem	Uterine atony	inadequate uterine tone; examples ’atony’, ’poor uterine tone’
	Laceration	laceration or tear, example ’lac’
	Uterine rupture	uterine rupture or uterine dehiscence, example ’uterine rupture’
	Retained products of conception	retained products of conception, retained placenta, or retained tissue, example ’rPOC’

Problem	Placenta accreta spectrum	an abnormally adherent placenta, or placenta accreta, percreta, or increta, example ’accreta’
	Abruption of the placenta	abruption or separation of the placenta before delivery, example ’abruption’
	Placenta previa	placenta previa, include ‘partial previa’ but only if present at delivery, do not include if resolved, example ’previa’
	Coagulation disorders	Coagulation disorders associated with a higher risk of bleeding, such as thrombocytopenia, HELLP, coagulation factor deficiency, and disseminated intravascular coagulopathy, which can be represented as ‘DIC’, ‘coagulopathy’ or ‘bleeding/oozing from all sites’
	PPH due to surgical causes	PPH due to surgical trauma during delivery; examples, ’an extension of the uterine incision’, ’laceration of uterine artery’
	Direct mention of PPH	Explicit documentation of postpartum hemorrhage or hemorrhage after delivery, example ’pph’
Delivery type	Cesarean delivery	Cesarean mode of delivery, example ’c-section’

Supplementary Table 1: List of postpartum hemorrhage (PPH)-related concepts that are extracted from obstetric discharge summaries and the descriptions for each concept provided to annotators. PPH, postpartum hemorrhage.

Concept (n)	Sensitivity	Specificity	PPV	Binary F1	Accuracy
Carboprost (n=49)	0.980	1.000	1.000	0.990	0.999
Laceration (n=421)	0.971	0.987	0.976	0.974	0.981
Bakri balloon (n=46)	0.957	0.999	0.978	0.967	0.997
Fresh frozen plasma (n=79)	1.000	0.994	0.919	0.958	0.994
Uterine atony (n=107)	0.888	0.999	0.990	0.936	0.989
Cesarean delivery (n=470)	0.915	0.965	0.945	0.930	0.945
Cryoprecipitate (n=29)	1.000	0.996	0.853	0.921	0.996
Methylergonovine (n=104)	0.865	0.998	0.978	0.918	0.986
Hysterectomy (n=55)	1.000	0.991	0.846	0.917	0.991
Placenta previa (n=170)	0.900	0.989	0.933	0.916	0.976
Misoprostol as a uterotonic (n=112)	0.982	0.972	0.786	0.873	0.973
O'Leary sutures (n=12)	0.833	0.999	0.909	0.870	0.997
Packed red blood cells (n=165)	0.909	0.965	0.811	0.857	0.957
Uterine rupture (n=5)	1.000	0.998	0.714	0.833	0.998
Direct mention of PPH (n=362)	0.732	0.986	0.960	0.831	0.908
Retained products of conception (n=102)	0.647	0.991	0.868	0.742	0.961
Placenta accreta spectrum (n=45)	0.667	0.992	0.769	0.714	0.980
Dilation and curettage (n=122)	0.648	0.971	0.718	0.681	0.937
Platelets (n=29)	0.862	0.983	0.556	0.676	0.980
PPH due to surgical causes (n=27)	0.407	0.999	0.917	0.564	0.986
Coagulation disorders (n=44)	0.364	0.997	0.842	0.508	0.974
Abruption of the placenta (n=47)	0.340	0.996	0.800	0.478	0.970
Manual extraction of placenta (n=110)	0.309	0.999	0.971	0.469	0.934

Concept (n)	Sensitivity	PPV	Note Accuracy
Estimated blood loss (n=212)	0.535	0.737	0.884

Supplementary Table 2: Performance of regular expressions assessed on 1,075 manually annotated discharge summaries. PPH, postpartum hemorrhage, PPV, positive predictive value.

Concept	Hospital	Count	Binary F1
PPH	BWH	195	0.891
	MGH	164	0.903
PPH due to surgical causes	BWH	11	0.381
	MGH	16	0.711
Placental abruption	BWH	38	0.872
	MGH	8	0.941
Cesarean section	BWH	331	0.963
	MGH	132	0.985
Misoprostol as a uterotonic	BWH	94	0.916
	MGH	17	0.971
Dilation and curettage	BWH	91	0.812
	MGH	29	0.846
Coagulation Disorders	BWH	35	0.870
	MGH	8	0.769
Fresh frozen plasma	BWH	74	0.953
	MGH	5	0.909
Carboprost	BWH	33	0.970
	MGH	15	0.846
Hysterectomy	BWH	42	0.955
	MGH	12	0.815
Laceration	BWH	258	0.984
	MGH	163	0.985
Manual extraction of placenta	BWH	86	0.740
	MGH	23	0.824
Methylergonovine	BWH	64	0.953
	MGH	39	0.974
Packed red blood cells	BWH	128	0.907
	MGH	37	0.850
Placenta accreta spectrum	BWH	37	0.846
	MGH	8	0.421
Placenta previa	BWH	121	0.940

	MGH	47	0.979
Retained products of conception	BWH	68	0.897
	MGH	32	0.933

Supplementary Table 3: Comparison of Flan-T5 Large Language Model (LLM) performance in extracting postpartum hemorrhage (PPH)-related concepts across Mass General Brigham hospital sites. All prompt engineering was performed exclusively on notes from Brigham and Women's Hospital, and the remaining hospitals serve as external test sets. Sites with at least 5 notes with the concept are included, and only labels with more than one site are shown. PPH, postpartum hemorrhage; BWH, Brigham and Women's Hospital, MGH, Massachusetts General Hospital.

Concept (n)	Sensitivity	Specificity	PPV	Binary F1	Accuracy
Laceration (n=54)	0.870	0.913	0.922	0.895	0.890
Bakri balloon (n=15)	1.000	1.000	1.000	1.000	1.000
Cesarean delivery (n=27)	1.000	1.000	1.000	1.000	1.000
Methylergonovine (n=32)	0.750	1.000	1.000	0.857	0.920
Placenta previa (n=2)	1.000	1.000	1.000	1.000	1.000
Fresh frozen plasma (n=14)	0.429	1.000	1.000	0.600	0.920
Carboprost (n=15)	0.667	1.000	1.000	0.800	0.950
Misoprostol as a uterotonic (n=17)	0.529	1.000	1.000	0.692	0.920
Hysterectomy (n=0)	N/A	N/A	N/A	N/A	N/A
O'Leary sutures (n=0)	N/A	N/A	N/A	N/A	N/A
Uterine atony (n=25)	0.640	1.000	1.000	0.780	0.910
Retained products of conception (n=19)	0.789	0.975	0.882	0.833	0.940
Direct mention of PPH (n=85)	0.929	1.000	1.000	0.963	0.940
Packed red blood cells (n=40)	0.950	0.917	0.884	0.916	0.930
Abruption of the placenta (n=2)	1.000	1.000	1.000	1.000	1.000
Coagulation disorders (n=2)	1.000	0.959	0.333	0.500	0.960
Cryoprecipitate (n=5)	1.000	0.989	0.833	0.909	0.990
Dilation and curettage (n=31)	0.419	0.942	0.765	0.542	0.780
Platelets (n=2)	1.000	0.959	0.333	0.500	0.960
Placenta accreta spectrum (n=2)	1.000	0.990	0.667	0.800	0.990
Manual extraction of placenta (n=13)	1.000	0.931	0.684	0.813	0.940
PPH due to surgical causes (n=5)	0.400	0.989	0.667	0.500	0.960
Uterine rupture (n=0)	N/A	N/A	N/A	N/A	N/A

Concept (n)	Sensitivity	PPV	Note Accuracy
Estimated blood loss (n=87)	0.757	0.813	0.780

Supplementary Table 4: Temporal generalizability of the zero-shot Flan-T5 model in identifying postpartum hemorrhage (PPH) concepts. We assessed the temporal generalizability of the Flan-T5 model by evaluating on 100 manually annotated notes with PPH ICD diagnostic codes from 2015-2022. We report the prevalence of each concept in the annotated evaluation set. We report model sensitivity, specificity, positive predictive value (PPV), binary F1 score, and accuracy for binary concepts, and we report sensitivity, PPV, and note-level accuracy in extracting the correct estimated blood loss values. N/A denotes labels without any positive examples in the labelled notes. PPH, postpartum hemorrhage, PPV, positive predictive value.

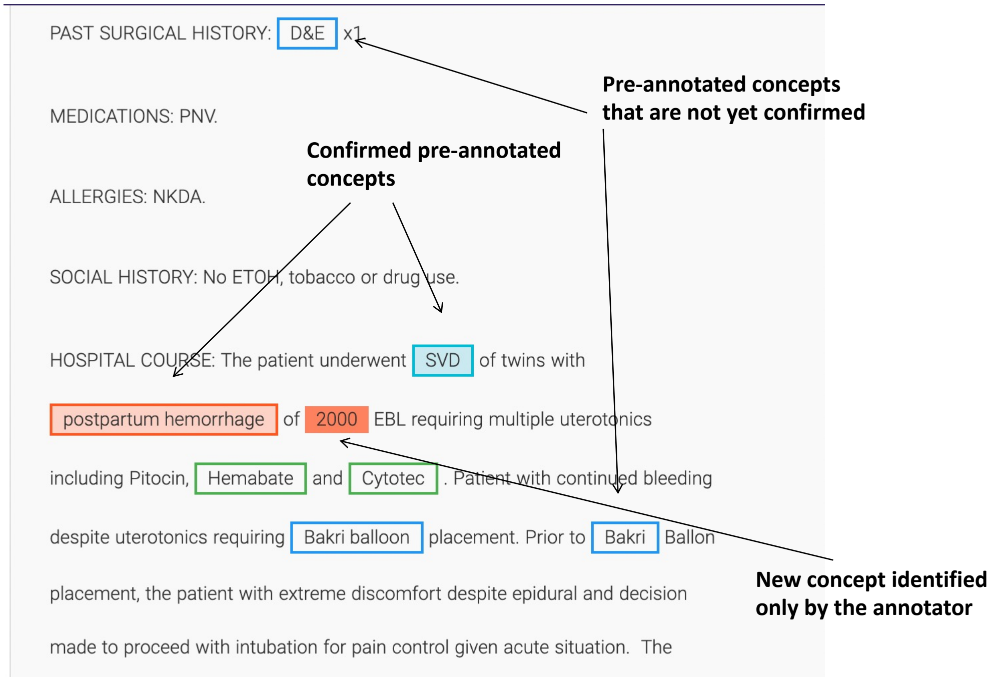

Supplementary Figure 1: An image capture of the modified PRAnCER annotation tool.

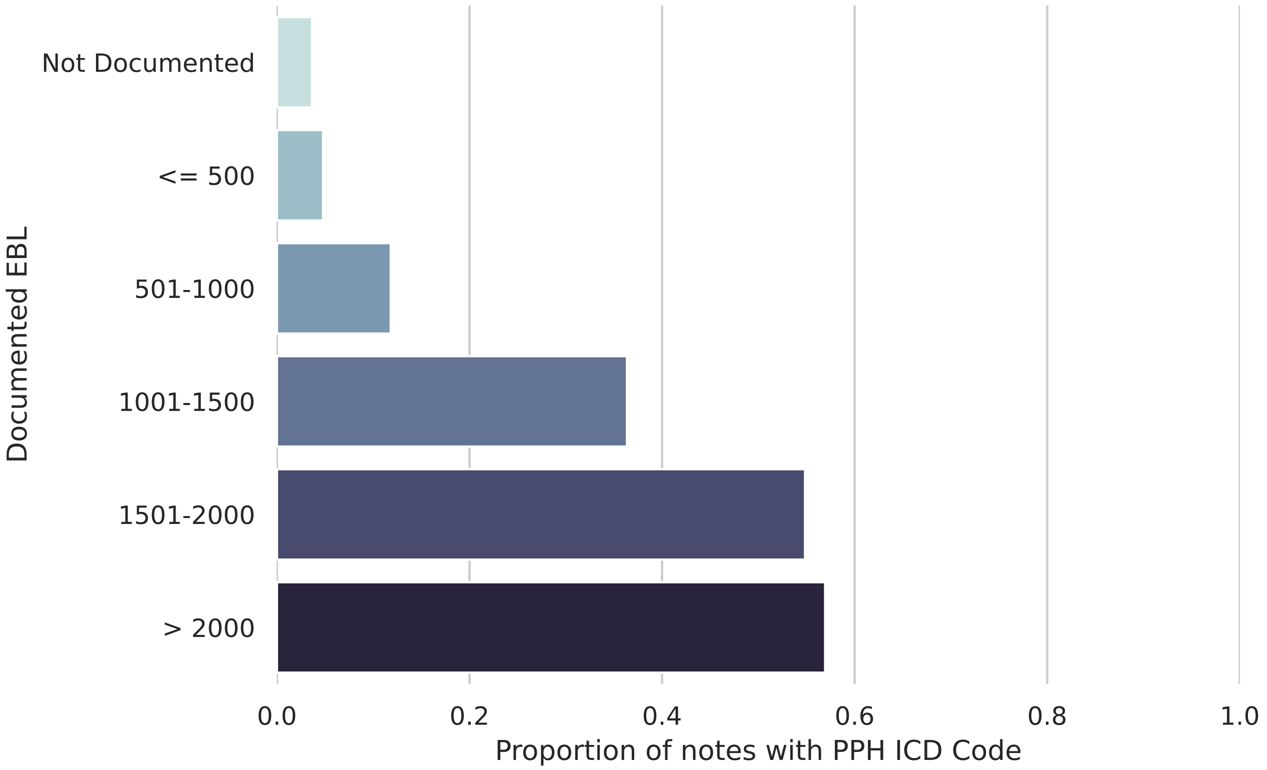

Supplementary Figure 2: Prevalence of postpartum hemorrhage(PPH) international classification of diseases (ICD) codes in notes with varying estimated blood loss values documented. The y-axis depicts the range of estimated blood loss (EBL) values extracted from the delivery note by Flan-T5 (if any), and the x-axis denotes the proportion of notes in each EBL category with a postpartum hemorrhage ICD diagnostic code. PPH ICD codes are defined according to the definition in Butwick et al. Refer to Figure 3 for a similar plot using the PPH ICD definition in Zheutlin et al. PPH, postpartum hemorrhage; ICD, international classification of diseases; EBL, estimated blood loss.

**Model**	**Sensitivity**	**Specificity**	**PPV**	**Binary F1**	**Accuracy**
Flan-T5 Zero Shot	0.986	0.857	0.947		**0.950**
Regex	**1.000**	0.643	0.878		0.900
ICD Code - Day of Note	0.472	**1.000**	**1.000**		0.620
ICD Code - Within 2 Days	0.847	0.964	0.984		0.880
ICD Code - Within 7 Days	0.944	0.857	0.944		0.920
ICD Code - Within 14 Days	0.972	0.857	0.946		0.940

Supplementary Table 5: Comparison of approaches for identifying discharge notes that describe a labor and delivery encounter on a random sample of 100 discharge summaries. ICD, international classification of diseases, PPV, positive predictive value.

Model Size	Sensitivity	Specificity	PPV	Binary F1	Accuracy
Small	0.833	0.136	0.116	0.204	0.220
Base	0.500	0.568	0.136	0.214	0.560
Large	0.333	**0.977**	0.667	0.444	0.900
XL	**1.000**	0.773	0.375	0.545	0.800
XXL	**1.000**	0.955	**0.750**	**0.857**	**0.960**

Supplementary Table 6: Impact of Flan T5 model size on zero shot classification performance for identifying mentions of postpartum hemorrhage in notes. All metrics are reported on the small prompt tuning set of 50 notes. PPV, positive predictive value.
